# The effect of nano silver fluoride, self-assembling peptide and sodium fluoride varnish on salivary cariogenic bacteria: a randomized controlled clinical trial

**DOI:** 10.1007/s00784-024-05562-0

**Published:** 2024-02-23

**Authors:** Sara M. Atteya, Hala A. Amer, Susan M. Saleh, Yara Safwat

**Affiliations:** 1 Department of Pediatric Dentistry and Dental Public Health, Faculty of Dentistry, Alexandria University, Champollion St, Egypt, Azarita, Alexandria 21527 Egypt; 2 Department of Medical Microbiology and Immunology, Faculty of Medicine, Alexandria University, Alexandria, Egypt

**Keywords:** Nanosilver fluoride, P11-4, Sodium fluoride, *Streptococcus mutans (S. mutans)*, *Lactobacilli*

## Abstract

**Objectives:**

To compare the antibacterial effect of Nanosilver Fluoride varnish (NSF) varnish, P11-4 and Sodium Fluoride (NaF) varnish against salivary *Streptococcus mutans (S. mutans) and Lactobacilli*.

**Methods:**

66 patients aged 10–24 years old were randomly assigned to receive single application of NSF, P11-4 or NaF varnish. Baseline unstimulated saliva samples were collected before the agents were applied and *S.mutans* and *Lactobacilli* colony forming units (CFU) were counted. After one, three and six months, microbiological samples were re-assessed. Groups were compared at each time point and changes across time were assessed. Multivariable linear regression compared the effect of P11-4 and NSF to NaF on salivary *S. mutans and Lactobacilli* log count at various follow up periods.

**Results:**

There was a significant difference in salivary *S. mutans* log count after 1 month between P11-4 (B= -1.29, *p* = 0.049) and NaF but not at other time points nor between NSF and NaF at any time point. The significant reduction in bacterial counts lasted up to one month in all groups, to three months after using P11-4 and NaF and returned to baseline values after six months.

**Conclusion:**

In general, the antimicrobial effect of P11-4 and NSF on salivary *S. mutans* and *Lactobacilli* was not significantly different from NaF varnish. P11-4 induced greater reduction more quickly than the two other agents and NSF antibacterial effect was lost after one month.

**Clinical relevance:**

NSF varnish and P11-4 have antimicrobial activity that does not significantly differ from NaF by 3 months. P11-4 has the greatest antibacterial effect after one month with sustained effect till 3 months. The antibacterial effect of NSF lasts for one month. NaF remains effective till 3 months.

**Trial Registration:**

This trial was prospectively registered on the clinicaltrials.gov registry with ID: NCT04929509 on 18/6/2021.

**Supplementary Information:**

The online version contains supplementary material available at 10.1007/s00784-024-05562-0.

## Introduction

Oral colonization by *S. mutans* is required for dental caries initiation, and *S. mutans* count higher than 10^5^ colony-forming unit (CFU)/ mL of saliva is an indicator of high caries risk [1]. *Lactobacilli* are highly acidogenic and aciduric and are cultured from established carious lesions [2]. *Lactobacilli* are secondary invaders whereas *S. mutans* are primary initiators of caries [3].

Silver nanoparticles (AgNPs) with 20 to 15,000 silver atoms and a diameter smaller than 100 nm have an antimicrobial activity at low concentration due to large surface-to-volume ratio. Silver has high affinity to compounds containing nitrogen, sulfur, and phosphorus leading silver to interact with the thiol groups of proteins and the phospholipid portion of the bacterial membrane [4], altering its permeability and causing its rupture and interfering with cell replication [4–6]. The addition of Nano-silver in varnishes can prevent dental plaque formation [7]. AgNPs, chitosan and fluoride were combined into Nano Silver Fluoride (NSF) demonstrating antimicrobial properties inhibiting *S.mutans* growth [8, 9] with no risk for living organisms or toxic effects to humans in vitro [10]. NSF did not show tissue darkening due to silver ion oxidation when in contact with the teeth [11]. Also, significant reduction in *S. mutans* count was reported in vivo after one and three months of using NSF in saliva and plaque [12].

Self-Assembling Peptide P11-4 (Curodont Repair) is a recent material used to prevent caries, made up of arginine, tryptophan, phenylalanine, glutamine, and glutamic acid. These amino acids undergo hierarchical self-assembly into fibrillar scaffolds in response to high ionic strength and acidic pH found in the caries lesion body thus guiding the regeneration of enamel tissue and promoting remineralization [13–15]. Moreover, arginine and tryptophan are extended, random-coil peptides with broad spectrum antimicrobial activity. When they come in contact with a membrane, they usually fold into amphipathic structures leading to membrane leakage. They also interact with intracellular bacterial components by inhibiting nucleic acid synthesis, protein production, or other enzyme activities as cell-wall synthesis [16]. The antibacterial efficacy can be considerably enhanced when peptides are self-assembled into nanostructures, and their stability can be enhanced because they become less sensitive to enzymatic degradation and uptake by the reticuloendothelial system [13–17].

A previous study has shown the antimicrobial effect of arginine and tryptophan as components present in self-assembling peptide [17]. Although previous literature has shown the remineralizing effect of P11-4 on early enamel caries lesions [18, 19], to the best to our knowledge no published studies have investigated whether P11-4 as a product has an antimicrobial effect on cariogenic salivary bacteria. Also, no previous clinical trials have shown whether NSF has an antibacterial effect that extends after three months. Assessing the antibacterial effect of these caries preventing agents helps understand their mode of action. In addition, determining the length of the time period over which the antibacterial effect, if present, exists, helps set the frequency of application of these agents. The aim of this study was to compare the antimicrobial effect of NSF varnish, P11-4 and NaF varnish after one, three and six months. The null hypothesis was that there will be no difference in the antimicrobial effect of the three agents.

## Materials and methods

### Ethical consideration and study design

This study was conducted to assess the secondary aim of a parallel randomized, three arm, clinical trial comparing the effect of NSF, P11-4 and NaF varnish on remineralizing early white spot caries lesions in young adults. The study was conducted from December 2020 to August 2022 in the Pediatric Dentistry clinic of the Faculty of Dentistry, Alexandria University. Microbiological analysis was conducted in the microbiology lab, Faculty of Medicine, Alexandria University. Ethical approval was obtained from the Research Ethics Committee, Faculty of Dentistry, Alexandria University (#0086 − 11/2019). The objectives, risks and benefits of the study were explained to patients and signed informed consent was obtained from participants older than 18 years of age and from guardians of participants aged 10 to 18 years before participating in the study. Also, participants younger than 18 years of age assented to participate in the study. The study was conducted in full accordance with the Helsinki declaration. The trial was registered at ClinicalTrials.gov NCT04929509. Patients were given oral hygiene instructions about brushing twice a day especially before bedtime. The importance of proper diet in preventing caries was explained in detail. Six tubes of fluoridated toothpaste (Signal 2 with 1450 PPm sodium fluoride) and a brush were provided to each participant on the day of the intervention to be used throughout the whole study period. Thus, the 6 tubes were given once/ at the same time and they were meant to last the whole duration of the study as part of the standard basic oral hygiene procedure and this was for all groups. Patients were referred for treatment in the faculty clinics to provide care beyond the study scope.

### Participants eligibility

Participants were enrolled if they were 10–24 years old which is the age of young adolescents and youth as described by the WHO [20], when there is high prevalence of non-cavitated caries lesions [21]. Participants were included if they had at least one visible white spot lesion (WSL) in the buccal surface of permanent teeth with ICDAS II score of 1, or 2 since bacteria colonize the demineralized areas of the tooth [22] and after they or their parents signed an informed consent form to participate in the study [23]. Patients were excluded if they received antibiotics within 1 month before saliva sample collection because it is a cause of dysbiosis and may affect bacterial growth in culture and if they had multiple cavitated carious lesions since salivary SM and LB counts are positively correlated with the decayed, missing and filled teeth (DMFT) score [24, 25].

### Sample size calculation

This study addressed the secondary outcome of a trial where the primary outcome was remineralization of early white spot caries lesions in young adults after P11-4, NSF and NaF varnishes [26]. The sample size of the original study was based on remineralization of white spot lesions after applying P11-4 and NaF varnish and 22 were needed per group. To ensure that the present study was adequately powered, we also calculated the sample size required to assess the antibacterial effect based on 95% confidence level and 80% study power. During the study planning, no estimates of the agents’ salivary antibacterial effects could be found from in vivo studies. Therefore, we used estimates from in vitro studies represented by the mean (SD) percentages inhibition of bacterial adsorption to enamel surface by NSF and NaF (88.94 (31.32) and 41.06 (50.76)) [27], respectively, because bacterial counts are correlated with bacterial adsorption to enamel [28, 29]. Sample size was calculated to be 14 participants per group. Thus, the number of participants required for the primary outcome (*n* = 20 per group, increased to 22 patients to make up for loss to follow up) would ensure adequate power to asses the secondary outcome addressed in the present study.

### Randomization, allocation concealment and blinding

**Fig. 1:**
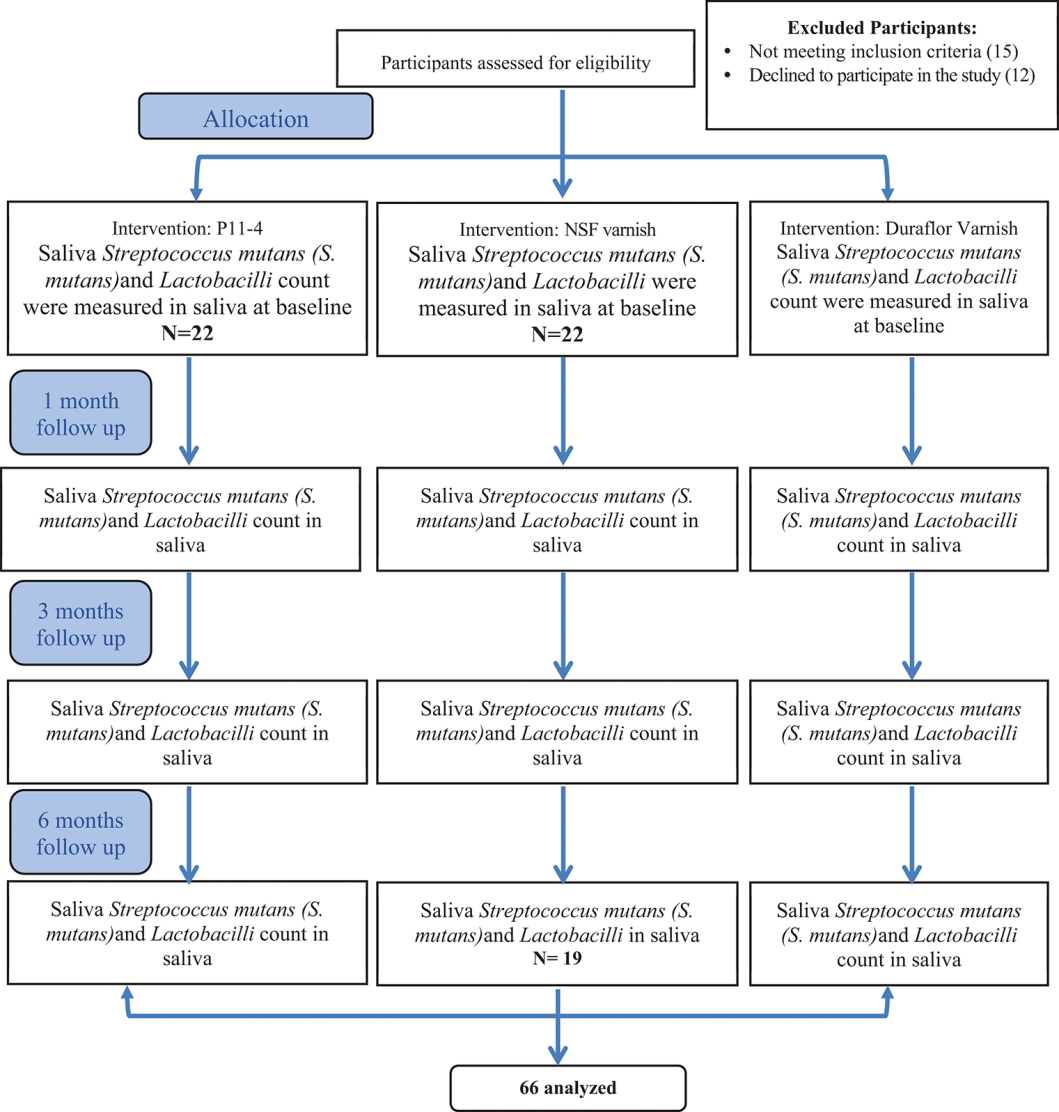
Participants flow chart

Participants were randomly and equally assigned using a computer generated list of random numbers to one of the three arms receiving P11-4 (Curodont Repair), NSF varnish and NaF varnish (Duraflor) (See Fig 1). Allocation was performed by a trial independent individual. Allocation was done in blocks of 3 to insure balanced groups [30]. The group to which each patient was allocated was written on identical sheets of paper, folded and placed inside opaque envelopes carrying the patient’s name. A trial independent person kept the envelopes secure and opened them only at the time of giving each patient the designated regimen. Participants were blinded to the agent they received by removing the labels from the bottles. The investigator could not be blinded since the method of application differed in the three agents.

### Intervention

NSF varnish was prepared in the Faculty of Pharmacy labs, Alexandria University, according to Wei et al’s. method [31]. The synthesis of aqueous solution of AgNPs was carried out via the chemical reduction of silver nitrate (1 mL, 0.11 M) with sodium borohydride (0.3 mL, 0.8 M) and chitosan biopolymer (28.7 mL, 2.5 mg/mL) as a stabilizing agent. Sodium fluoride (10,147 ppm of fluorine) was incorporated at the end of the experiment.

After partial isolation with cotton rolls and saliva ejector, each tooth with WSL in the NSF group received two drops of NSF applied with a micro brush, equivalent to 10 mg of the solution [31]. The NSF solution was left in contact with the tooth surface for 2 min.

The P11-4 was applied on the WSL following the manufacturers’ instructions. The tooth was isolated using rubber dam then cleaned using sodium hypochlorite 2% for 20 s. The WSL was etched with phosphoric acid gel 35–37% for 20 s, then rinsed with water and dried. Then the Curodont Repair was applied on the tooth surface [32].

The third group received 5% NaF varnish (Duraflor^®^). After partial isolation, the teeth with WSL were dried before the application of a very thin coat of the varnish, which was then allowed to dry for 10 s. The varnish hardens on contact with saliva or moisture [25]. Each agent was applied once at baseline.

### Outcome assessment

Saliva samples were collected at baseline before the agents were applied, and after one, three and six months.

Saliva collection was done in the morning (9–11 am) and patients were asked to rinse their mouth with water five minutes before sampling. This was done in all study groups and repeated before each saliva sample was taken. No prophylaxis was done at home except brushing with the fluoridated toothpaste distributed to them. The following procedure was followed to collect whole saliva according to Fejerskov and Thylstrup [33]: The patient was instructed not eat or drink (except water) one hour before saliva collection, samples were collected at the same time of day for all participants, and the patient was seated in a relaxed position in an ordinary chair.

Unstimulated whole saliva samples [34, 35] were collected continuously for 5 min in sterile containers which were sealed immediately. The patient was asked to drool out saliva in a sterile container and 2- 3 ml of unstimulated whole saliva were collected. The containers were labelled and stored in an ice box set below 4^o^C. Microbiological analyses were done within 45 min after sample collection [12]. Mitis Salivarius Agar (MSA) supplemented with potassium tellurite and Rogosa SL were used as culture media for isolation of salivary *S. mutans* and *Lactobacilli*, respectively. Saliva (1 mL) was diluted to 1:10 dilution by adding 9 mL of sterile saline solution and tenfold serial dilutions of saliva were made up to 10^− 6^ before plating. Ten microliters of each dilution were spread on the surface of the plates, placed in an anaerobic jar with AnaeroGas Pack system and incubated at 37 °C for 4–5 days [30]. Counting of colonies was done automatically using TotalLab™ 1D software. Then, the count of the colonies was calculated by multiplying the number of colonies on the plate by the dilution factor expressed as the number of colony forming units per milliliter (CFU/mL) of saliva [30, 31]. The outcome variables were the log counts of *S. mutans* and *Lactobacilli* at baseline, 1, 3 and 6 months. Participants’ oral health practices such as use of fluoridated toothpaste and sugar consumption was calculated as quantitative variable based on the frequency of daily consumption of any sugar containing food or drink at least once per day and their socioeconomic background (age, sex) were recorded as confounders [36].

### Statistical analysis

Median and Inter Quartile Range (IQR) were used for descriptive statistics. Log counts were calculated. Intention to treat analysis was used. The groups were compared regarding absolute and log bacterial count at each follow-up period using Kruskal Wallis test. Differences across time within each group with post-hoc comparison were assessed using Friedman test. Two multivariate linear regression models were developed to assess the effect of P11-4 and NSF compared to NaF on salivary *S. mutans* and *Lactobacilli* log count at baseline, 1, 3 and 6 months adjusting for age, sex, sugar intake frequency, using fluoridated toothpaste, and the number of white spot lesions. We also calculated the adjusted mean salivary *S. mutans* and *Lactobacilli* log counts in each group at each follow-up period. Significance level was set at *P* < 0.05. All tests were two tailed. Data were analyzed using SPSS for Macintosh version 28 [37]. (See Fig. 1).

## Results

The study included 66 patients at baseline. After 3 months, the drop out was 13.6% in the NSF group, 9.1% in the P114 group and 18.2% in the NaF group. After 6 months, the drop out in both the NSF and P11-4 groups was 13.6% and in the NaF group was 22.7%. The demographic characteristics of study participants are presented in Table 1. There were no statistically significant differences among the study groups except in the frequency of sugar intake (*P* = 0.031) with greater frequency of sugar intake in the NSF group.

**Table 1:** Sample description for the study groups

		NSF (*N* = 22 patients)	P11-4 (*N* = 22 patients)	NaF (*N* = 22 patients)	P value
Age: Mean (SD)		11.73 (2.87)	12.55 (3.54)	13.77 (4.6)	0.196
Sex: n (%)	Male	7 (31.8%)	9 (40.9%)	8 (36.4%)	0.822
	Female	15 (68.2%)	13 (59.1%)	14 (63.6%)	
Frequency of sugar intake: Mean (SD)		2.45 (1.34)	1.5 (1.01)	2.09 (1.19)	0.031*
Frequency of brushing with fluoridated toothpaste	Never	5 (22.7%)	2 (9.1%)	4 (18.2%)	0.477
	More than 1 time per month	3 (13.6%)	4 (18.2%)	7 (31.8%)	
	Once or twice a week	10 (45.5%)	8 (36.4%)	5 (22.7%)	
	Once per day	2 (9.1%)	6 (27.3%)	3 (13.6%)	
	Twice per day	2 (9.1%)	2 (9.1%)	3 (13.6%)	
DMFT: Mean (SD)		0.86 (1.42)	0.86 (1.67)	1.82 (2.79)	0.458
Number of teeth with white spot lesions: Mean (SD)		2 (1.45)	2 (1.35)	2.68 (1.36)	0.177

*Statistically significant *p* value<0.05

Table 2 shows the bivariate comparison of salivary *S. mutans* absolute and log counts among the three groups at different follow up periods. There were no significant differences among groups except after one month (*P* = 0.019) where P11-4 group had lower count than the other two groups. There were significant differences in counts across time per group (*P* < 0.001), with significant reductions from baseline to one and three months in each group, and to 6 months only in the NaF group (*P* = 0.006).

**Table 2:** Comparison of salivary *S. mutans* levels among the study groups at baseline and after one, three- and six-months in bivariate analysis

		NSF (*N* = 22 patients, *n* = 44 lesions)	P11-4 (*N* = 22 patients, *n* = 44 lesions)	NaF (*N* = 22 patients, *n* = 59 lesions)	P value
		Median (IQR)			
Baseline	Count	10 (7.55) × 10^4^	1 (1.08) × 10^5^	1 (0.9) × 10^5^	0.513
	Log 10	5 (0.47)	5 (0.5)	5 (0.75)	
1 month	Count	2.14 (7.75) × 10^3^	0 (1.11 × 10^3^)	1 (4.15) × 10^4^	0.019*
	Log 10	3.29 (3.88)	0 (3.05)	4 (4.62)	
3 months	Count	1.3 (7.6) 8 × 10^4^	6.94 (5.9) × 10^4^	1 (2.93) × 10^4^	0.475
	Log 10	4.1 (1.54)	3.84 (4.77)	4 (4.40)	
6 months	Count	5 (11.5) × 10^4^	8.05 (19.3) × 10^4^	1 (0.95)× 10^5^	0.489
	Log 10	4.70 (0.93)	4.89 (1.32)	4 (1.27)	
***P*** **value**		< 0.001*	< 0.001*	< 0.001*	
**Pairwise comparisons**	***P*** ^***a***^	< 0.001*	0.001*	< 0.001*	
	***P*** ^***b***^	0.005*	0.007*	< 0.001*	
	^***Pc***^	0.521	0.701	0.006*	

*Statistically significant *p* value<0.05

**P**^**a**^ : comparison between baseline and 1 month, **P**^**b**^ : comparison between baseline and 3 months, **P**^**c**^ : comparison between baseline and 6

Table 3 shows the bivariate comparison of salivary *Lactobacilli* absolute and log counts among the three groups at different time points. There were no significant differences among the three groups at any time point (*P* > 0.05). There were statistically significant within group differences in each of the three groups (*P* < 0.001) across time. The reduction from baseline to one and three months (*P* < 0.001) was significant in each group whereas the count increased after six months values so that there were no differences between baseline and six months values in NSF, P11-4 and NaF (*P* = 0.52, 0.35, and 0.22 respectively).

**Table 3:** Comparison of salivary *Lactobacilli* levels among the study groups at baseline and after one, three and six months in bivariate analysis

		NSF (*N* = 22 patients, *n* = 44 lesions)	P11-4 (*N* = 22 patients, *n* = 44 lesions)	NaF (*N* = 22 patients, *n* = 59 lesions)	P value
		Median (IQR)			
Baseline	Count	1 (2.08) × 10^5^	1.8 (2.14) × 10^5^	1 (1.1) × 10^5^	0.513
	Log 10	5 (0.52)	5.25 (0.5)	5 (0.32)	
1 month	Count	1 (2.35) × 10^4^	1 (8.35) × 10^4^	1 (8.05) × 10^4^	0.693
	Log 10	4 (1.12)	4 (4.92)	4 (2.01)	
3 months	Count	5 (11.8) × 10^4^	3.3 (7.17)× 10^4^	1.1 (6) × 10^4^	0.195
	Log 10	4.69 (1.08)	4.52 (0.93)	4.04 (0.83)	
6 months	Count	13 (21.27) × 10^4^	7.35 (24.33) × 10^4^	9 (17.88) × 10^4^	0.380
	Log 10	5.11 (0.61)	4.86 (0.86)	4.95 (1.27)	
***P*** **value**		< 0.001*	< 0.001*	< 0.001*	
**Pairwise comparisons**	***P*** ^***a***^	< 0.001*	< 0.001*	< 0.001*	
	***P*** ^***b***^	< 0.001*	< 0.001*	< 0.001*	
	***P*** ^***c***^	0.521	0.35	0.22	

*Statistically significant *p* value<0.05

**P**^**a**^ : comparison between baseline and 1 month, **P**^**b**^ : comparison between baseline and 3 months, **P**^**c**^ : comparison between baseline and 6 months

Table 4 shows the multivariable linear regression assessing the effect of P11-4 and NSF compared to NaF on salivary *S. mutans* log count at various follow up periods with adjustment for confounders. After 1 month, the P11-4 group had significantly lower log count than NaF (B= -1.29, 95%CI: -2.57, -0.01, *P* = 0.049) whereas NSF had lower log count than NaF with no significant difference (B= -0.52, 95%CI: -1.78, 0.74, *P* = 0.41). Compared to NaF, the log counts of P11-4 and NSF at three and six months were higher but not significantly different (*p* > 0.05).

**Table 4:** Multivariable linear regression assessing the effect of P11-4 and NSF compared to NaF on salivary *S. mutans* log count at various follow up periods

	One month		Three months		Six months	
	B (95% CI)	P value	B (95% CI)	P value	B (95% CI)	P value
P11-4 vs. NaF	-1.29 (-2.57, -0.01)	0.049*	0.18 (-1.18, 1.53)	0.79	0.09 (-0.46, 0.64)	0.75
NSF vs. NaF	-0.52 ( -1.78, 0.74)	0.41	0.88 (-0.45, 2.21)	0.19	0.14 (-0.40, 0.69)	0.60

*Statistically significant *p* value<0.05

Models are adjusted for age, sex, DMFT, sugar frequency, frequency of brushing with fluoridated toothpaste, number of white spot lesions

The dependents also include baseline log count (not shown)

Table 5 shows the multivariable linear regression assessing the effect of P11-4 and NSF compared to NaF on salivary *Lactobacilli* log count at various follow up periods with adjustment for confounders. The reduction in the salivary *Lactobacilli* log count was noticed only in the P11-4 group after 1 and 6 months. The log count of the P11-4 group was higher than the NaF after 3 months and so were the log counts of the NSF group after 1, 3 and 6 months although none of these differences were significant.

**Table 5:** Multivariable linear regression assessing the effect of P11-4 and NSF compared to NaF on salivary *Lactobacilli* log count at various follow up periods

	One month		Three months		Six months	
	B (95% CI)	P value	B (95% CI)	P value	B (95% CI)	P value
P11-4 vs. NaF	-0.27 (-1.54, 0.99)	0.67	0.08 (-0.93, 1.08)	0.88	-0.01 (-0.42, 0.41)	0.98
NSF vs. NaF	0.24 ( -1.01, 1.49)	0.70	0.55 (-0.44, 1.54)	0.27	0.19 (-0.21, 0.60)	0.35

Models are adjusted for age, sex, DMFT, sugar frequency, frequency of brushing with fluoridated toothpaste, number of white spot lesions

The dependents also include baseline log count (not shown)

**Fig. 2:**
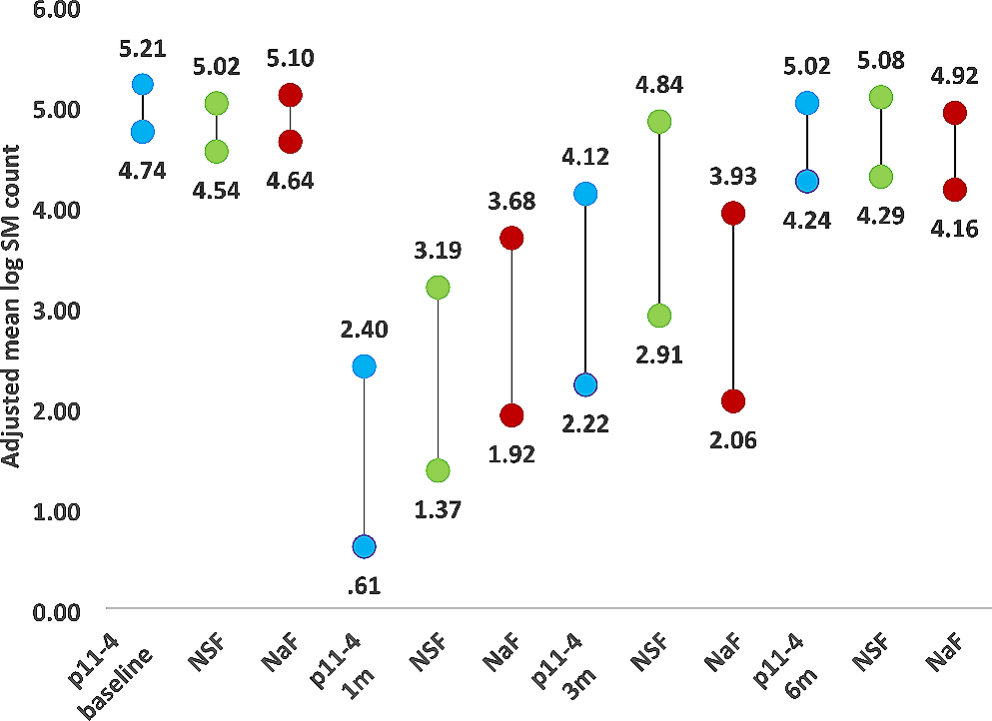
The Adjusted mean log *S. mutans* count of P11-4, NSF and NaF at various time intervals

**Fig. 3:**
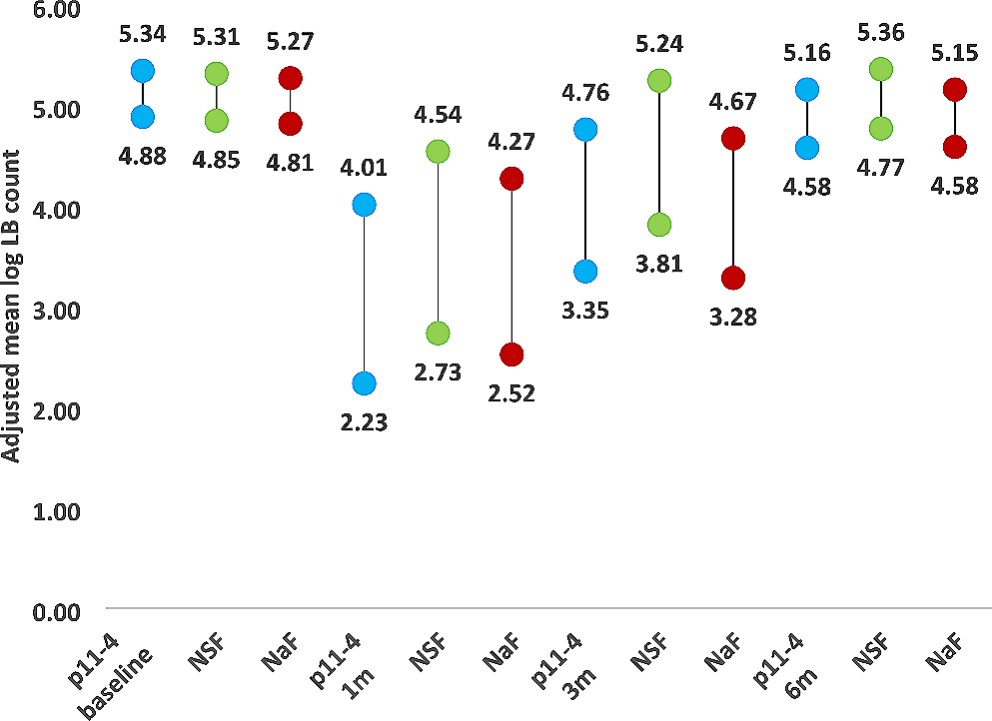
showing the Adjusted mean log *Lactobacilli* count of P11-4, NSF and NaF at various time intervals

Figures 2 and 3 show the estimated marginal means of salivary *S. mutans* and *Lactobacilli* log count in the P11-4, NSF and NaF groups at various follow up periods. After one month, there was a significant drop in the salivary *S. mutans* and *Lactobacilli* log count in the three groups. The significant drop continued to 3 months only in the p11-4 and NaF groups but not the NSF group. After 6 months the salivary *S. mutans* and *Lactobacilli* log count returned back to baseline values in the three groups.

## Discussion

The adjusted analysis showed that there was a significant difference in only *S.mutans* log count between P11-4 and NaF after one month with P11-4 having lower count although this count increased steadily afterwards so that there were no significant differences in the two micro-organisms counts among the groups at any other time point. The antibacterial effect was sustained up to one month in the three groups and remained till three months in the P11-4 and NaF groups only. Thus, there is no support to reject the null hypothesis of the study.

In the present study, there were no significant differences in the two micro organisms counts between NSF and NaF at any time point. A possible explanation for this no significant difference is that although the nanosilver particles in NSF have antimicrobial effect, its fluoride content in it, 10,147 ppm [3], is less than that in the NaF varnish, 22,600 ppm [38]. This may explain the superior antibacterial effect of NaF leading to the longest period of reduced *S. mutans* count till 6 months. Clinical trials investigating the antibacterial effect of NSF varnish are rare. However, our findings agree with a clinical trial showing no significant difference between NSF and NaF in salivary *S. mutans* count after three months of one application of both agents in children aged 8–10 years old [12].

Our findings showed significant difference in salivary *S. mutans* count after 1 month between the P11-4 and the NaF groups. This agrees with a recently published in vitro study [39] that showed significantly greater median inhibition zone of *S. mutans* by fluoridated P11-4 than NSF and NaF after 24 h. Despite the difference in the duration of follow up, the similarity supports our conclusion about the initial greater antibacterial effect of P11-4 than other fluoride agents although this effect is not sustained beyond one month.

In this study, we observed that the antibacterial effect of NSF lasted for only one month. This agrees with some invitro studies reporting that the effect of NSF against *S. mutans* remained only till one month [7, 27, 40, 41]. Also, Fujun et al. [42] reported that the antimicrobial effect of the highest concentrations of nanosilver containing glass ionomer cement against *S. mutans* was lost after 2 months. Our findings also showed a significant reduction in *Lactobaccilli* log count after one month. Although no clinical trial has investigated the antimicrobial effect of NSF varnish against salivary *Lactobacilli*, invitro studies proved the antimicrobial effect of NSF particles against *Lactobacilli* [43–45] and an animal study [46] showed a significant reduction in salivary *Lactobacilli* count in rats after 24 h of the application of nanoparticles incorporated in an orthodontic adhesive. Thus, our study fills a knowledge gap by providing clinical evidence showing that the antimicrobial effect of NSF varnish against salivary *S. mutans* and *Lactobacilli* lasts for one month.

Our results have shown that P11-4 significantly decreased salivary *S. mutans* and *Lactobacilli* counts till three months. To the best of our knowledge there are no published clinical studies investigating the antimicrobial effect of P11-4. This antimicrobial effect may be attributed to the amino acids arginine and tryptophan [16]. The antibacterial effect is enhanced when peptides are self-assembled in nanostructures [10, 20]. Another explanation could be due to the possible decrease of enamel surface roughness after the application of P11-4 as reported in previous research [47] since *S. mutans* adhere more on rough surfaces [48, 49]. Thus, our clinical trial fills another knowledge gap about the duration of the antimicrobial effect of P11-4 on salivary *S. mutans* and *Lactobacilli.*

Our study fills a knowledge gap by providing evidence on the antibacterial effect of P11-4 and NSF compared to NaF at various follow-up intervals and on how long this effect can be sustained after a single application of each agent which has clinical implications. The adjusted analysis showed that the counts of both types of micro organisms returned to baseline values after 6 months indicating that the antibacterial effect is expected to last for less than 6 months.

Our study is limited by using salivary samples to assess bacterial counts. Future studies may be needed to confirm our findings using dental plaque samples. However, previous studies [50, 51] reported a positive correlation between cariogenic bacterial levels in plaque and saliva. Also, we collected unstimulated saliva similar to previous studies [34, 35] assessing salivary bacterial colonies by culture. The collection of stimulated or unstimulated saliva may affect the results of microbiological analysis only when 16 S rRNA pyrosequencing was performed [52] with limited impact in culture based studies.

## Conclusions

*S. mutans* and *Lactobacilli* are the main bacterial strains responsible for dental caries, therefore assessing and comparing the amount and duration of the antibacterial effect of the tested materials would provide opportunities for their use as anticaries products. The findings demonstrated the antimicrobial effect of both NSF and P11-4 on salivary *S. mutans* and *Lactobacilli* which did not generally differ from NaF varnish. The antimicrobial effects of all agents peak at 1 month then salivary *S. mutans* and *Lactobacilli* counts return back to baseline levels after 6 months.

### Electronic supplementary material

Below is the link to the electronic supplementary material.

Supplementary Material 1

## Data Availability

The datasets used and/or analysed during the current study are available from the corresponding author on reasonable request.

## References

[1] Bratthall D, Carlsson P (2021) Clinical microbiology of saliva. In: Tenovuo JO (ed) Human saliva: clinical chemistry and microbiology. CRC, pp 203–241

[2] Guo L, Wenyuan S (2013). Salivary biomarkers for caries risk assessment. J Calif Dent Assoc.

[3] Badet C, Thebaud NB (2008). Ecology of lactobacilli in the oral cavity: a review of literature. Open Microbiol J.

[4] Durán N, Marcato PD, Conti RD, Alves OL, Costa F, Brocchi M (2010). Potential use of silver nanoparticles on pathogenic bacteria, their toxicity and possible mechanisms of action. J Braz Chem Soc.

[5] Panacek A, Kvítek L, Prucek R (2006). Silver colloid nanoparticles: synthesis, characterization, and their antibacterial activity. J Phys Chem B.

[6] Martínez-Castañon GA, Nino-Martinez N, Martinez-Gutierrez F, Martínez-Mendoza JR, Ruiz F (2008). Synthesis and antibacterial activity of silver nanoparticles with different sizes. J Nanopart Res.

[7] Haghgoo R, Saderi H, Eskandari M, Haghshenas H, Rezvani M (2014). Evaluation of the antimicrobial effect of conventional and nanosilver-containing varnishes on oral streptococci. J Dent (Shiraz)‎.

[8] Targino AG, Flores MA, dos Santos Junior VE, de Godoy Bené Bezerra F, de Luna Freire H, Galembeck A, Rosenblatt A (2014). An innovative approach to treating dental decay in children. A new anti-caries agent. J Mater Sci Mater Med.

[9] Nanda KJ, Naik S (2020). An in-vitro comparative evaluation of pre-treatment with nano-silver fluoride on inhibiting secondary caries at tooth restoration interface. Cureus.

[10] Freire PL, Albuquerque AJ, Farias IA et al (2016) Antimicrobial and cytotoxicity evaluation of colloidal chitosan–silver nanoparticles–fluoride nanocomposites. Int J Biol Macromol 93(Pt A 896–903. 10.1016/j.ijbiomac.2016.09.052 10.1016/j.ijbiomac.2016.09.052 27642129

[11] Santos VE, Filho V, Targino A (2014). A new silver-bullet to treat caries in children–nano silver fluoride: a randomised clinical trial. J Dent.

[12] Waikhom N, Agarwal N, Jabin Z, Anand A (2022). Antimicrobial effectiveness of Nano Silver Fluoride Varnish in reducing Streptococcus mutans in saliva and plaque biofilm when compared with chlorhexidine and Sodium Fluoride varnishes. J Clin Exp Dent.

[13] Carrick LM, Aggeli A, Boden N, Fisher J, Ingham E, Waigh TA (2007). Effect of ionic strength on the self-assembly, morphology and gelation of pH responsive β-sheet tape-forming peptides. Tetrahedron.

[14] Aggeli A, Bell M, Boden N, Carrick LM, Strong AE (2003). Self-assembling peptide polyelectrolyte beta-sheet complexes form nematic hydrogels. Angew Chem Int Ed Engl.

[15] Li R, Guo W, Yang B (2011). Human treated dentin matrix as a natural scaffold for complete human dentin tissue regeneration. Biomaterials.

[16] Nguyen LT, Haney EF, Vogel HJ (2011). The expanding scope of antimicrobial peptide structures and their modes of action. Trends Biotechnol.

[17] Hong W, Zhao Y, Guo Y (2018). PEGylated self-assembled Nano-Bacitracin A: probing the Antibacterial mechanism and real-time tracing of target delivery in vivo. ACS Appl Mater Interfaces.

[18] Wierichs RJ, Carvalho TS, Wolf TG (2021). Efficacy of a self-assembling peptide to remineralize initial caries lesions - a systematic review and meta-analysis. J Dent.

[19] Keeper JH, Skaret LJ, Thakkar-Samtani M (2022). Systematic review and Meta-analysis on the Effect of Self-assembling peptide P11-4 on initial caries lesions. medRxiv.

[20] World Health Organization (WHO) (2019). Child and adolescent health and development.

[21] Zafar M, Levy SM, Warren JJ, Xie XJ, Kolker J, Pendleton C (2022). Prevalence of non-cavitated lesions and progression, regression, and no change from age 9 to 23 years. J Public Health Dent.

[22] Abou Neel EA, Aljabo A, Strange A, Ibrahim S, Coathup M, Young AM, Bozec L (2016). Demineralization-remineralization dynamics in teeth and bone. Int J Nanomed.

[23] Gözetici B, Öztürk-Bozkurt F, Toz-Akalın T (2019). Comparative Evaluation of Resin Infiltration and Remineralisation of Noncavitated smooth surface caries lesions: 6-month results. Oral Health Prev Dent.

[24] Sounah SA, Madfa AA (2020). Correlation between dental caries experience and the level of Streptococcus mutans and lactobacilli in saliva and carious teeth in a Yemeni adult population. BMC Res Notes.

[25] Jing D, Hao J, Shen Y, Tang G, Lei L, Zhao Z (2019). Effect of fixed orthodontic treatment on oral microbiota and salivary proteins. Exp Ther Med.

[26] Atteya SM, Amer HA, Saleh SM, Safwat Y (2023). Self-assembling peptide and nano-silver fluoride in remineralizing early enamel carious lesions: randomized controlled clinical trial. BMC Oral Health.

[27] Teixeira JA, Silva AVCE, Dos Santos Júnior VE (2018) Effects of a New Nano-Silver Fluoride-Containing Dentifrice on Demineralization of Enamel and Streptococcus mutans Adhesion and Acidogenicity. Int J Dent 2018: 1351925. 10.1155/2018/1351925 10.1155/2018/1351925 PMC5964412 29853891

[28] Mummolo S, Nota A, Albani F (2020). Salivary levels of Streptococcus mutans and lactobacilli and other salivary indices in patients wearing clear aligners versus fixed orthodontic appliances: an observational study. PLoS ONE.

[29] Testa M, Ruiz de Valladares R, Benito de Cárdenas IL (1999). Correlation between bacterial counts in saliva and subgingival plaque. Acta Odontol Latinoam.

[30] Saghaei M (2004). Random allocation software for parallel group randomized trials. BMC Med Res Methodol.

[31] Wei D, Sun W, Qian W, Ye Y, Ma X (2009). The synthesis of Chitosan-based silver nanoparticles and their antibacterial activity. Carbohydr Res.

[32] Curodont (2019) ^™^ Biomedical products for tooth preservation. https://www.curodont.com/wpcontent/uploads/2018/09/CURODONTREPAIR_M958_EN_V2.0.pdf. Accessed 26 June 2019

[33] Fejerskov O, Thylstrup A, Mitchell DA, Mitchell L (1994). The oral environment and introduction. Textbook of Clinical Cariology.

[34] Chokshi A, Mahesh P, Sharada P, Chokshi K, Anupriya S, Ashwini BK (2016). A correlative study of the levels of salivary Streptococcus mutans, lactobacilli and Actinomyces with dental caries experience in subjects with mixed and permanent dentition. J Oral Maxillofac Pathol.

[35] Liu JF, Hsu CL, Chen LR (2019). Correlation between salivary mutans streptococci, lactobacilli and the severity of early childhood caries. J Dent Sci.

[36] World Health Organization (WHO) (2013). Oral health surveys: basic methods.

[37] IBM Corp (2021). IBM SPSS Statistics for Machontosh, Version 28.0.

[38] Chu CH, Lo E (2008). Uses of sodium fluoride varnish in dental practice. Ann R Australas Coll Dent Surg.

[39] Elkaddah RK, Attia NM, Elwassefy N, Abdellatif AM (2024). Antimicrobial efficacy of Nano Silver Fluoride, fluoridated self-assembling peptide and Sodium Fluoride varnishes on oral Streptococcus Mutans. Egypt Dent J.

[40] Jasso-Ruiz I, Velazquez-Enriquez U, Scougall-Vilchis RJ, Morales-Luckie RA, Sawada T, Yamaguchi R (2020). Silver nanoparticles in orthodontics, a new alternative in bacterial inhibition: in vitro study. Prog Orthodont.

[41] Hernández-Sierra JF, Ruiz F, Pena DC (2008). The antimicrobial sensitivity of Streptococcus mutans to nanoparticles of silver, zinc oxide, and gold. Nanomedicine.

[42] Li F, Li Z, Liu G, He H (2013). Long-term antibacterial properties and bond strength of experimental nano silver-containing orthodontic cements. J Wuhan Univ Technol-Mater Sci Ed.

[43] Azarsina M, Kasraei S, Yousef-Mashouf R, Dehghani N, Shirinzad M (2013). The antibacterial properties of composite resin containing nanosilver against Streptococcus mutans and Lactobacillus. J Contemp Dent Pract.

[44] Tian X, Jiang X, Welch C (2018). Bactericidal effects of Silver nanoparticles on Lactobacilli and the underlying mechanism. ACS Appl Mater Interfaces.

[45] Ahmed O, Sibuyi NRS, Fadaka AO (2022). Antimicrobial effects of Gum Arabic-Silver nanoparticles against oral pathogens. Bioinorg Chem Appl.

[46] Bahador A, Ayatollahi B, Akhavan A, Pourhajibagher M, Kharazifard MJ, Sodagar A (2020). Antimicrobial Efficacy of Silver Nanoparticles Incorporated in an Orthodontic Adhesive: An Animal Study. Front Dent.

[47] Magalhães GAP, Fraga MAA, de Souza Araújo IJ, Pacheco RR, Correr AB, Puppin-Rontani RM (2022). Effect of a self-assembly peptide on Surface Roughness and hardness of bleached enamel. J Funct Biomater.

[48] Eslemez Topcu E, Şahin O, Köroğlu A, Cömert F, Yilmaz B (2022). Surface roughness and Streptococcus mutans adhesion on surface sealant agent coupled interim crown materials after dynamic loading. BMC Oral Health.

[49] Soliman WE, Ali AI, Elkhatib WF (2019). Evaluation of surface roughness and Streptococcus mutans adhesion to bulk-fill resin composites polished with different systems. Adv Microbiol.

[50] Zoitopoulos L, Brailsford SR, Gelbier S, Ludford RW, Marchant SH, Beighton D (1996). Dental caries and caries-associated micro-organisms in the saliva and plaque of 3- and 4-year-old afro-caribbean and caucasian children in south London. Arch Oral Biol.

[51] Nguyen M, Dinis M, Lux R, Shi W, Tran NC (2022). Correlation between Streptococcus mutans levels in dental plaque and saliva of children. J Oral Sci.

[52] Gomar-Vercher S, Simón-Soro A, Montiel-Company JM, Almerich-Silla JM, Mira A (2018). Stimulated and unstimulated saliva samples have significantly different bacterial profiles. PLoS ONE.

